# A Multi-Scale Feature Fusion Method Based on U-Net for Retinal Vessel Segmentation

**DOI:** 10.3390/e22080811

**Published:** 2020-07-24

**Authors:** Dan Yang, Guoru Liu, Mengcheng Ren, Bin Xu, Jiao Wang

**Affiliations:** 1 Key Laboratory of Data Analytics and Optimization for Smart Industry (Northeastern University), Ministry of Education, Shenyang 110819, China; 2 Key Laboratory of Infrared Optoelectric Materials and Micro-Nano Devices, Shenyang 110819, China; 3 College of Information Science and Engineering, Northeastern University, Shenyang 110819, China; ren_mengcheng@163.com (M.R.); wangjiao@ise.neu.edu.cn (J.W.); 4 School of Computer Science and Engineering, Northeastern University, Shenyang 110819, China; xubin@mail.neu.edu.cn

**Keywords:** multi-scale, retinal vessel segmentation, U-Net, inception structure, max-pooling index

## Abstract

Computer-aided automatic segmentation of retinal blood vessels plays an important role in the diagnosis of diseases such as diabetes, glaucoma, and macular degeneration. In this paper, we propose a multi-scale feature fusion retinal vessel segmentation model based on U-Net, named MSFFU-Net. The model introduces the inception structure into the multi-scale feature extraction encoder part, and the max-pooling index is applied during the upsampling process in the feature fusion decoder of an improved network. The skip layer connection is used to transfer each set of feature maps generated on the encoder path to the corresponding feature maps on the decoder path. Moreover, a cost-sensitive loss function based on the Dice coefficient and cross-entropy is designed. Four transformations—rotating, mirroring, shifting and cropping—are used as data augmentation strategies, and the CLAHE algorithm is applied to image preprocessing. The proposed framework is tested and trained on DRIVE and STARE, and sensitivity (Sen), specificity (Spe), accuracy (Acc), and area under curve (AUC) are adopted as the evaluation metrics. Detailed comparisons with U-Net model, at last, it verifies the effectiveness and robustness of the proposed model. The Sen of 0.7762 and 0.7721, Spe of 0.9835 and 0.9885, Acc of 0.9694 and 0.9537 and AUC value of 0.9790 and 0.9680 were achieved on DRIVE and STARE databases, respectively. Results are also compared to other state-of-the-art methods, demonstrating that the performance of the proposed method is superior to that of other methods and showing its competitive results.

## 1. Introduction

Retinal fundus images facilitate the study of various structures in the retina [1]. The morphological changes of the retinal blood vessels are closely related to fundus diseases such as glaucoma, age-related macular degeneration, and diabetic retinopathy [2]. Therefore, accurate segmented images of retinal blood vessels can assist experts in early diagnosis and monitoring of the above diseases, thus preventing blindness [3]. However, retinal blood vessels are difficult to segment completely, manual labeling is time-consuming and labor-intensive, and there is a large amount of subjectivity [4]. Therefore, a lot of research has been done to achieve automatic segmentation of retinal vessels [5,6]. At present, retinal blood vessel automatic segmentation technology has become an important tool for clinical medical disease screening and diagnosis. In addition, the technology can also provide people living in remote areas with advanced medical technologies, equivalent to those in developed areas, which improves people’s health and quality of life.

Many retinal vascular segmentation methods have been proposed in recent years, which can be divided into supervised and unsupervised methods, according to whether prior information is required or not. Unsupervised methods do not require a priori labeling information, and use the similarity between data for analysis. They can be subdivided into five sub-categories: matched filtering, morphological processing, vessel tracking, multi-scale analysis and model-based algorithms [7]. Based on the matched filter method, the retinal image is convolved with the two-dimensional Gaussian kernel function, and the retinal blood vessel image is obtained by extracting the maximum response of the Gaussian matched filter in different directions. Chaudhuri et al. [8] were primarily worked on matched filter approach and find that cross-section greyscale profile and intensity similarity of vessels follows the curve form of Gauss. However, the detection accuracy of the technique is very low. Hoover et al. [9] described an automated retinal blood vessel segmentation method using local and global vessel features cooperatively on matched filter response images. Miri and Mahloojifar [10] presented a method to detect retinal blood vessels effectively using curvelet transform and multistructure elements morphological processing. The edges of the retinal image were enhanced by modifying the curvelet transform coefficients, where the erroneously detected edges were deleted during the modification process, so that tiny vessels can be better segmented. Wang et al. [11] proposed a comprehensive method combined matched filtering with multiwavelet kernels and multiscale hierarchical decomposition for retinal vessels segmentation. The method can be directly used on different data sets without preprocessing and training. The experimental results demonstrated an excellent performance, but its calculation was expensive. Mendonca and Campilho [12] presented an automated method for detection of the retinal blood vessels by combining differential operators to extract vessel centerlines and morphological filters. The results approximate the accuracy of expert manual labelling, and the sensitivity and specificity do not drop significantly. However, the method is very penalizing for larger vessels. Based on the continuous morphology nature of blood vessels, the vascular tracking algorithm firstly establishes an initial seed node on the vascular structure, and then tracks along the direction of blood vessels from the node to stop when the morphology is not continuous, so as to find the vascular structure between different initial nodes. The center of the longitudinal section of the blood vessel was determined by greyscale intensity and flexura. This type of method can provide highly accurate vessel widths, but they cannot segment the retinal vessels without seed points [13]. Vlachos and Dermatas [14] implemented a multi-scale retinal vessel segmentation method. The algorithm combined a multiscale line-tracking procedure and a morphological post-processing. Experimental results demonstrate that the algorithm is robust even in the case of a low signal-to-noise ratio, but its drawback is the high misclassification rate of fundus optic discs. Although the unsupervised methods performed well in the detection of retinal vessels according to the structure of vessels without using a priori labeling information, the effectiveness on thin tiny vessels and low contrast images still has a lot of room for improvement [15]. 

Compared with the unsupervised methods, supervised methods takes the manually marked image as the training data label and generate the corresponding algorithm model. The process of supervised retinal vessel segmentation methods include two steps: (1) feature extraction and (2) classification. For different segmentation tasks, the k-nearest neighbor classifier [16], support vector machine classifier [17], convolutional neural network architecture [18] and other segmentation methods are proposed. Fraz et al. [19]. presented an ensemble method for segmentation of retinal blood vessel based on the line strength measures and orientation analysis of the gradient vector field, and a boosted decision tree classifier was applied in this method, but no objective analysis was given. Orlando et al. proposed an extensive description based on a fully connected conditional random field model, where a structured output support vector machine is applied to learned the parameters. However, most existing methods are based on the ground truth of manual segmentation, which is easily affected by subjective factors and pathological areas. [20]. In recent years, deep learning has made great breakthroughs in various fields of computer vision [21]. The mainstream algorithm of deep learning, convolutional neural networks, is widely used in image classification, target recognition and natural language processing [22]. In 2015, Ronneberger et al. in [23] presented a U-Net network for biomedical image segmentation, which used multi-level skip connection and encoder-decoder structure to improve the accuracy of pixel localization and captured context features. Aiming at solving the boundary detection problem, a deep learning architecture utilizing a fully-connected Conditional Random Fields and a full convolutional neural network was proposed in [24] to extract retinal vessel. Diego et al. [25] classified retinal vascular pixels by a supervised method. This method is based on a network structure, and computes the feature vector composed of gray-level and moment invariants for model training. Mo and Zhang [26] developed a deeply neural network by fusing multilevel full convolutional network and incorporating auxiliary supervision at some intermediate layers for vessel segmentation more robustly. Jiang et al. [27] proposed an automatic segmentation model for retinal vessels by D-Net, and used dilated convolution, pre-processing and denoising. The network can obtain denser feature information and alleviate the excessive loss of feature information of tiny vessels. Despite the great success of neural network-based methods, due to the small diameter of the thin tiny vessel and the poor contrast of the fundus image, existing methods still cannot accurately segment the capillaries.

Considering the existing problem of retinal vessel segmentation, we proposed a multi-scale feature fusion retinal vessel segmentation method based on U-Net, which integrated data augmentation, data preprocessing, and MSFFU-Net model. The model introduced an inception structure [28] into the multi-scale feature extraction encoder. The max-pooling index [29] was applied during the upsampling process in the feature fusion decoder part. Then, the network contained two skip connections: one was that each set of feature maps generated on the encoder path were concatenated to the corresponding feature maps on the decoder path; the other was that transferring of max pooling indices instead of the whole values from the encoder to the decoder. Moreover, we designed a cost-sensitive loss function based on the Dice coefficient and cross entropy. Rotating, mirroring, shifting and cropping were used as data augmentation strategies, and the CLAHE algorithm was applied to data preprocessing. The proposed method was trained and tested on public datasets DRIVE and STARE, and sensitivity, specificity, accuracy and area under curve (AUC) were adopted as evaluation metrics. Comparisons with U-Net model verified the effectiveness and robustness of the proposed model. The experimental results show that the method can solve the problem of insufficient segmentation of multiscale blood vessels. Compared to other state-of-the-art methods including the unsupervised and supervised methods, the results demonstrated that the performance of the proposed method is superior to existing methods and shows its competitive. 

The contributions of our work can be elaborated as follows:

(1) We propose a multi-scale feature fusion retinal vessel segmentation method based on U-Net.
(2) Four transformations—rotating, mirroring, shifting and cropping—are used as data augmentation strategies to improve the generalization ability of the proposed method.
(3) We design a cost-sensitive loss function based on the Dice coefficient and cross entropy, which improves the classification effect of categories with a small sample number.

The rest of this paper is organized as follows: Section 2 presents the improved method; Section 3 analyzes and discusses the experimental results; Section 4 summarizes the paper and draws our conclusions.

## 2. Proposed Method 

### 2.1. U-Net Model

**Figure 1:**
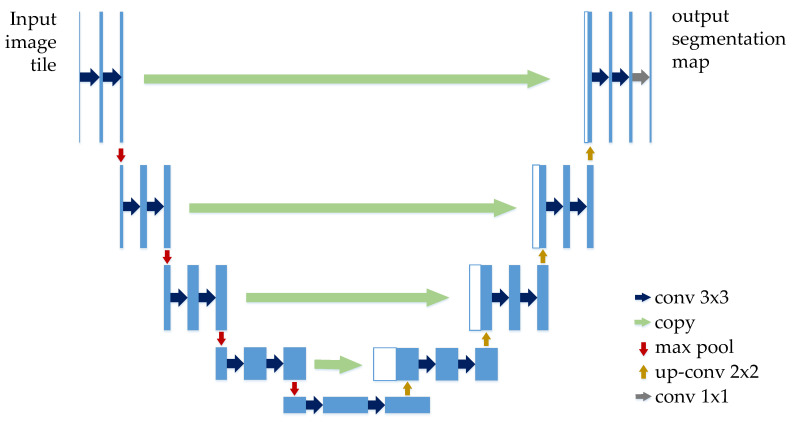
The classic U-Net architecture.

U-Net is a convolutional network for biological image segmentation proposed by Ronneberger et al. in 2015 [23]. The network structure is symmetrical and mainly consists of an encoder part (left side) and a decoder part (right side) as shown in Figure 1. The encoder part follows the typical architecture of a convolutional network to extract spatial features from images. It involves the repeated application of two 3 × 3 convolutions, each followed by an activation function (ReLU) and a max-pooling operation with a pooling size of 2 × 2 and step size of 2 for down-sampling. The number of repetitions is four. In each down-sampling step, we double the number of feature channels. On the other hand, the decoder is applied to construct a segmentation map based on the features obtained from the encoder. It includes an up-sampling of the 2 × 2 transpose convolution of the feature map, which reduces the feature channel by half, a connection with the correspondingly feature map in the encoder path, and two 3 × 3 convolutions, each followed similarly by a activation function ReLU. In the last layer, a 1 × 1 convolution is used to generate the final segmentation map.

### 2.2. MSFFU-Net

**Figure 2:**
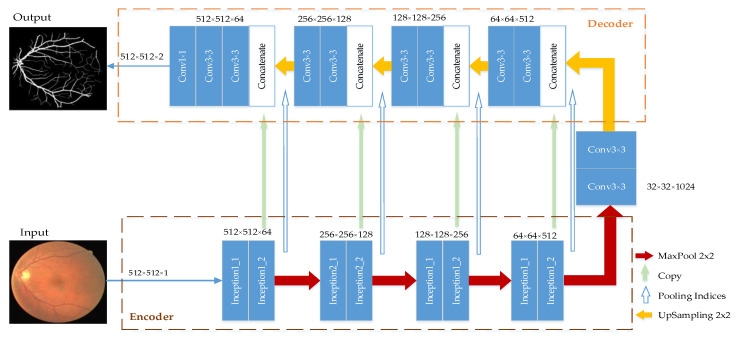
MSFFU-Net structure.

The U-Net module realizes the fusion of the low-level image features and the high-level image features through the multi-level jump structure, which can extract more sufficient features. We proposed a multi-scale feature fusion retinal vascular image segmentation model based on U-Net, which consisted of multi-scale feature extraction encoder and feature fusion decoder, named MSFFU-Net. The 3 × 3 convolution operation in U-Net was replaced by the inception structure to extract as much information as possible about the retinal microvessels in the encoder part of the improved network. The multi-scale feature extraction module based on Inception adopted the convolution of different kernel sizes, which can enhance the generalization and expressiveness of the network. Then, in order to accurately retain the location information of the object features, the max-pooling index was applied during the upsampling process in the feature fusion decoder part. The overall structure is shown in Figure 2.

#### 2.2.1. Multi-Scale Feature Extraction Encoder

**Figure 3:**
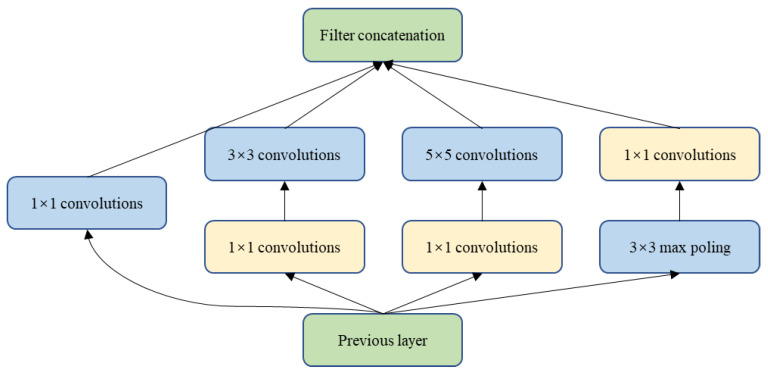
The basic inception module.

This part consists of the repeated application of two multi-scale feature extraction modules and a max-pooling operation with a pooling size of 2 × 2 and step size of 2 for downsampling. The number of repetitions is four. In each step of downsampling, we double the number of feature channels. The Inception module [28] is the core structure of the GoogleNet network model that achieved the best results in ILSVRPC 2014. The Inception network’s architecture improves the utilization of computing resources within the network by increasing the depth and width of the network while keeping the computing budget constant. Filters of different sizes are employed in the same layer to handle the feature information of multiple scales, and then the features are aggregated in the next layer so that the fusion features of multiple scales can be extracted in the next Inception module [30]. The basic inception structure uses filters of sizes 1 × 1, 3 × 3, and 5 × 5, as shown in Figure 3. The convolution output and the max pool output are connected to a single output vector to form the input for the next stage. Rather than using a properly sized filter at one level, using multiple sized filters makes the network wider and deeper, so it can recognize different scale features. The resulting feature maps are concatenated and then go to the next layer. Further refinement is achieved by applying the 1 × 1 convolution operation to merge dimensional reduction before the convolution of 3 × 3 and 5 × 5.

**Figure 4:**
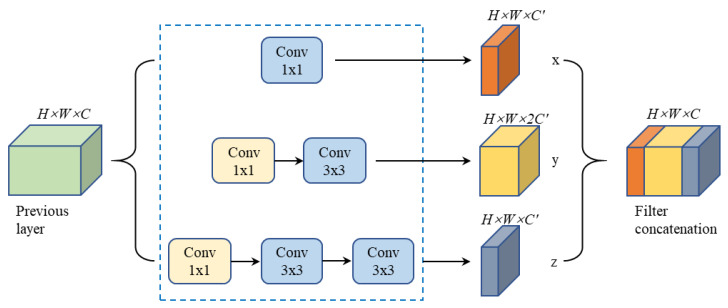
Multi-scale feature exception module based on an inception structure.

In this work, we used two 3 × 3 filters in series instead of a 5 × 5 convolution filter because they have an equivalent receiving field. This reduced the computational cost, resulting in a reduction in the number of parameters and a faster training speed. The multi-scale feature extraction module based on inception structure is shown as Figure 4. We perform three sets of different scale convolution operations (1 × 1 convolution, 3 × 3 convolution and two 3 × 3 convolutions) on the previous layer feature map (H × W × C), with a step size of 2 and a padding of 0. The other two 1 × 1 convolution operations play the role of compressing the number of channels and reducing the calculation cost. In the multi-scale feature extraction module designed in this paper, the feature images obtained by the 3 × 3 convolution account for half of the total feature images, and the remaining two convolutions each account for one quarter. The three sets of feature maps (x, y, z) are concatenated to be the back layer feature map. Furthermore, we also adopted an activation function (leaky ReLU) and a batch normalization (BN) [31] following each convolutional layer. The activation function introduces nonlinear characteristics into the network and maps the input to the output. The leaky ReLU function is simple to calculate, can solve the problem of gradient disappearance and gradient explosion, and can also effectively solve the gradient death of ReLU. BN could help our algorithm achieve high-speed coverage and alleviate the problem of overfitting. Different kernel sizes for convolution operations result in different receptive fields, which allows the model to incorporate multi-scale feature maps and has good learning ability for target features of various sizes.

#### 2.2.2. Feature Fusion Decoder

**Figure 5:**
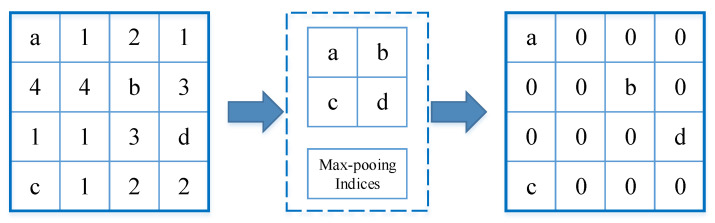
Max-pooling indices storage module. a, b, c, d correspond to values in a feature map and represent the maximum value in the 2 × 2 region respectively.

In the improved decoder part, the structural framework of the original U-Net decoder was retained. It included an upsampling of the 2 × 2 transpose convolution of the feature map, which reduced the feature channel by half, a connection with the correspondingly feature map in the multi-scale feature extraction encoder path, and two 3 × 3 convolutions, each followed similarly by an activation function leaky ReLU. At the last layer, a 1 × 1 convolution was used to generate the final segmentation map. In addition, we added a max-pooling index storage module [29], as shown in Figure 5. The pooling process in the encoder used max-pooling and recorded the max value indices. The upsampling process was based on the index values recorded during the pooling. In the feature map, the non-index position was filled with 0, and the corresponding pixel value was filled in the index position. 

**Figure 6:**
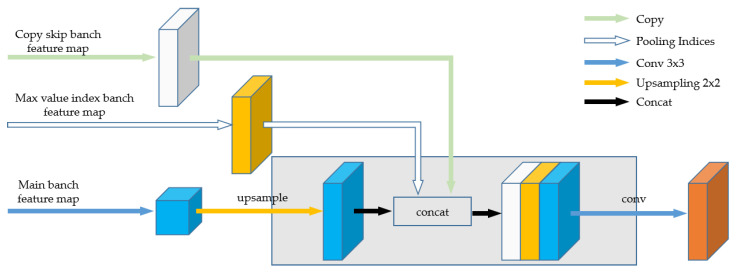
The combined operations (upsampling, copy, max value index, and convolution) involved while merging feature maps from the extended skip modules with the decoder.

As a whole, each block in the feature fusion decoder was also a repeating structure of up-sampling, followed by multiple 3 × 3 deconvolutions, Batch Normalization (BN), and leaky ReLU activation operations. Simultaneously, the MSFFU-Net contained extended two skip connections: one was that each set of feature maps generated on the encoder path are concatenated to the corresponding feature maps on the decoder path; the other was that transferring of max pooling indices values from the encoder to the decoder to locate contour position information of multi-scale retinal vessel features for higher segmentation accuracy [32]. The feature maps of the upsampling operation were merged with the corresponding output feature maps of the two extended skip modules [33], as shown in Figure 6.

### 2.3. Loss Function Design

The quality of segmenting retinal vessels using the proposed method depends not only on the choice of model architecture, but also on the loss function chosen for training the model and optimizing network parameters. The loss function (also known as the error function) reflects the difference between the predicted value and the ground truth. The pixels can be categorized into vessel and background; the statistics show that only 10% of pixels in the fundus image are blood vessels. The ratio of blood vessels to background pixels is very uneven [34]. If the characteristics of the fundus image are not fully considered in the process of designing the loss function, the learning process will tend to segment the background region. The learning process will fall into a local minimum of the loss function, and vascular pixels are often lost or only partially identified. In the work, we propose a novel loss function based on the Dice coefficient and cross entropy, and added a cost-sensitive matrix to the cross-entropy loss function.

The Dice loss function that is very popular in medical image segmentation is defined as:

(1) $$Loss_{Dice}=1-\frac{2|A\cap B|}{\left|A|+|B\right|}$$

In Equation (1), A represents the fundus blood vessel region segmented by the algorithm, and B denotes the fundus blood vessel region manually segmented by the expert. |A∩B| represents the same area of the retinal blood vessel region segmented by the proposed method and expert. Additionally, the equation shows that if the Dice coefficient is close to 1, the prediction result will be close to the ground true value. The cross-entropy loss function is defined as follows:

(2) $$Loss_{cross\_entropy}=-\sum \left[\begin{array}{cc} y & 1-y \end{array}\right]\left[\begin{array}{c} \log(p)\\ \log(1-p) \end{array}\right]$$

where y and p are the ground truth and prediction, respectively y is 0 or 1 and p is between 0 and 1. The cost-sensitive matrix M is incorporated, as shown in formula (3), this can avoid under-fitting due to the small number of retinal blood vessels during the neural network learning process [35]. When retinal blood vessels are misclassified, the cost will be greater and the attention to retinal blood vessels will be increased:

(3) $$M=\left[\begin{array}{cc} 1 & 6\times I(p<=0.5)\\ 0 & 1 \end{array}\right]$$

In the matrix, 1 at the (1,1) position is the penalty coefficient for predicting the blood vessel type as the blood vessel type; 6 at the (1,2) position is the penalty coefficient for predicting the blood vessel category as the background category; 0 at the (2,1) position is no penalty for predicting the background category as the blood vessel category; 1 at the (2,2) position represents the penalty coefficient for predicting the background type as the background type. In the neural network training, we did not adjust for the class-imbalance based on the number of retinal pixels against the background. The penalty coefficient for predicting the blood vessel category as the background category is always 6. The indicator function *I*(*p* ≤ 0.5) is denoted as:

(4) $$I(p<=0.5)=\{\begin{array}{cc} 1, & p<=0.5\\ 0, & p>0.5 \end{array}$$

The improved cross-entropy loss function is expressed as:

(5) $$Loss_{cross\_entropy}=-\sum \left[\begin{array}{cc} y & 1-y \end{array}\right]\left[\begin{array}{cc} 1 & 6\times I(p<=0.5)\\ 0 & 1 \end{array}\right]\left[\begin{array}{c} y\log(p)+(1-y)\log(1-p)\\ y\log(p)+(1-y)\log(1-p) \end{array}\right]$$

Considering m samples $\left\{\left(x_{1},y_{1}\right),\left(x_{2},y_{2}\right)\cdots \left(x_{m},y_{m}\right)\right\}$, the overall cost function of the MSFFU-Net model can be defined as:

(6) $$J(W,b,x,y)=\frac{1}{m}\sum_{k=1}^{m}\left[\alpha Loss_{Dice}(y_{k},p_{k})+(1-\alpha )Loss_{cross\_entropy}(y_{k},p_{k}\right)]$$

In this formula, W and b represent the parameters obtained by training in the model, m represents the total number of samples, y represents the label value of the sample, and x represents the input value of the model. α is the weighting coefficient between Dice and the cross-entropy loss function, which can be obtained by solving the partial derivatives of W and b for the objective function J(W,b,x,y) using the backpropagation algorithm. The value ranges from 0 to 1. In the neural network model, the larger the value of W, the more serious the overfitting phenomenon. Therefore, L2 regularization item was added in Equation (6). The equation was rewritten as:

(7) $$J(W,b)=J(W,b,x,y)+\frac{\lambda }{2}\sum W^{2}$$

where λ is the regularization coefficient. This approach can obviously speed up the convergence of the network. The proposed model parameters are converged to the optimal value after a certain number of iterations.

### 2.4. Dataset

The comparison of evaluation indexes of retinal vessel segmentation algorithm requires a standard data set. 

**Figure 7:**
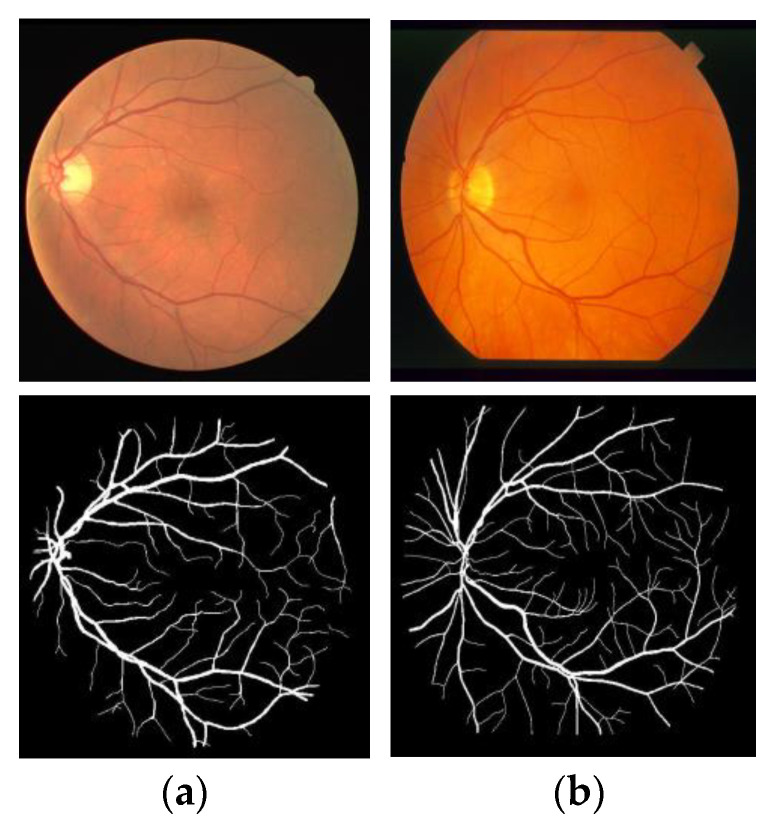
Examples of DRIVE dataset and STARE dataset. (**a**) Original image; (**b**) Ground truth. First row represents original image and second row represents ground truth image.

The published standard datasets for retinal images are DRIVE and STARE, which are often used in the research of retinal vessel segmentation methods. Figure 7 shows an example of the two datasets.

#### 2.4.1. DRIVE

The DRIVE dataset was composed by Niemeijer’s team in The Netherlands in 2004 from a diabetic retinopathy screening program [16]. The 40 samples were selected from 400 diabetic patients aged between 25 and 90 years. Among them, 33 cases did not show the characteristics of diabetic retinopathy, and seven cases did show them. The dataset was randomly divided into two groups, one was the test set and the other was the training set, each group contained twenty images. The images were captured by a CR53 camera (Canon, Oita, Japan) and the size of each image was 565 × 584. There were two manual segmentation result of experts in the test set. Among the results of one expert segmentation, the blood vessels pixels accounted for 12.7% of the total pixels, while the other expert was 12.3%. The former is called set A, while the latter is called set B. The manual segmentation result in the set A in DRIVE is used as the standard reference image. 

#### 2.4.2. STARE

This paper also tested the images in the STARE dataset. The STARE dataset was compiled and published by Hoover et al. in 2000 [9]. It includes 20 retinal images, of which 10 are healthy images and the rest are retinopathy images. Retinal photographs, each 700 × 605 in size, were taken by a Topcon-50 (Mitsui Bussan Electronics, Tokyo, Japan) fundus camera. Similarly, the images are randomly divided into 10 training images and 10 test images. There were also two manual segmentation result of experts in the dataset. Among the results of the one expert, blood vessels accounted for 10.4% of the total pixels, while the other expert accounted for 14.9. In this paper, the segmentation result of the first expert in STARE is used as the standard reference image.

### 2.5. Data Augmentation

**Figure 8:**
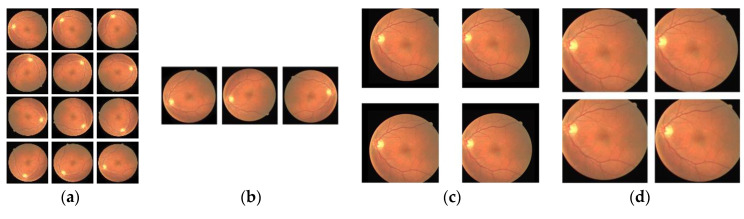
The four steps for dataset augmentation. (**a**) Rotating; (**b**) Mirroring; (**c**) Shifting; (**d**) Cropping.

Data augmentation is widely applied in convolutional neural networks because of its high efficiency and operability. In this paper, the training sets in DRIVE and STARE are used as the training data of the model. A total of 30 original retinal vascular images were included. Considering that the number of dataset is too small, the model will be prone to overfitting and has a poor classification performance. Therefore, it is necessary to augment the dataset for achieving the better results. And at the same time, data augmentation is an effective solution to equip a convolutional neural network with the invariance and robustness. Four image processing steps were used for augmenting dataset and they are rotating, mirroring, shifting and cropping. The detail process was as shown in Figure 8. First, each image was rotated at 30-degree intervals. Then, a mirror flip operation on each image was performed. Next, each image was randomly shifted 20 to 50 pixels towards its four corners. Finally, each image was randomly cut four times, and the size of the patch was 512 × 512. 

### 2.6. Data Preprocessing

**Figure 9:**
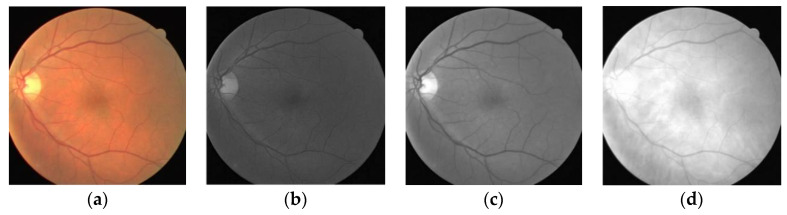
Retinal R, G and B channel images. (**a**) DRIVE dataset fundus image, (**b**) R channel image, (**c**) G channel image, (**d**) B channel image.

The original retinal blood vessel image has a low contrast. The features of retinal blood vessel are not obvious. In order to improve the performance of the MSFFU-Net model, the technique that enhances image contrast was used to make the retinal blood vessel features more obvious [3]. The use of several monochromatic representations of the original color images was explored in order to evaluate their adequacy for the segmentation of the retinal blood vessels. According to Figure 9b–d, the green channel of RGB retinal image has highest contrast between the blood vessels and background. The green channel as a natural choice for the segmentation of the retinal blood vessels have been considered in several research works [12,16,36]. Therefore, the green channel is used for further processing and MSFFU-Net training treatment. 

**Figure 10:**
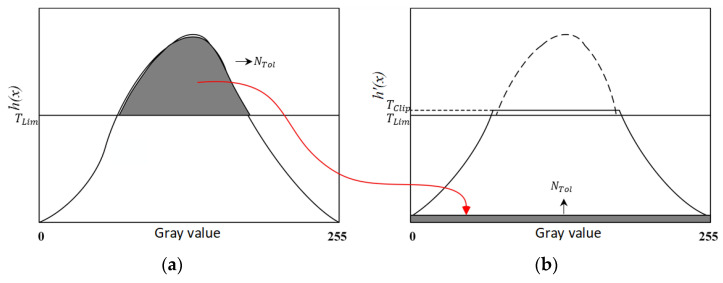
Schematic diagram of the CLAHE algorithm. (**a**) The original image, (**b**) Redistributed by CLAHE.

In addition to extracting the green channel of the retinal image, further processing is needed to make the features of the blood vessels more prominent. The Contrast Limiting Adaptive Histogram Equalization (CLAHE) algorithm can improve the contrast between blood vessels and background while suppressing noise [37]. We implemented the CLAHE algorithm to get a well-contrasted image. First, the original retinal blood vessel image was evenly divided into small sub-blocks of the same size. Then perform a histogram height limit on each sub-block, and the histogram was equalized for each sub-block, as shown in Figure 10. Finally, the transformed gray value was obtained by interpolation operation for each pixel, thereby realizing contrast-limited adaptive image enhancement.

The specific process of CLAHE algorithm is as follows:

(1) Divide into multiple sub-blocks. The retinal blood vessel image was divided into sub-block of the same size and not overlapping. Each sub-block containing the number of pixels as M. The larger the sub-block, the more obvious the enhancement, but the more the image details were lost. In the proposed method, set M = 8 × 8. The size of the original image was 512 × 512, and the size of each sub-block was 64 × 64.
(2) Calculate the histogram. Calculate the histogram distribution h(x) of each sub-block, where x is the gray value of each pixel in the image, ranging from 0 to 255.
(3) Calculate the shear threshold $T_{Clip}$. As shown in Equation (8):
  (8) $$T_{Clip}=\frac{M\times N}{L}+[\alpha \times (M\times N-\frac{M\times N}{L})]$$
  where α is the normalized clipping coefficient, whose value ranges from 0 to 1. The smaller the value, the better the effect. In this paper, the value is 0.05.
(4) Redistribution of pixels. Count the number of pixel points above the threshold as $N_{Tol}$, and calculate the increment of gray value of each pixel as $N_{Ace}=\frac{N_{Tol}}{L}$. Finally, the assigned threshold as $T_{Lim}=T_{Clip}-N_{Ace}$:
  (9) $$h^{\prime }(x)=\{\begin{array}{c} T_{Clip}\begin{array}{cc} & h(x)>T_{Lim} \end{array}\\ h(x)\begin{array}{cc} & h(x)\leq T_{Lim} \end{array} \end{array}$$
(5) Pixel gray value reconstruction. Bilinear interpolation was used to reconstruct the gray value of the center pixel of each row molecule block.

**Figure 11:**
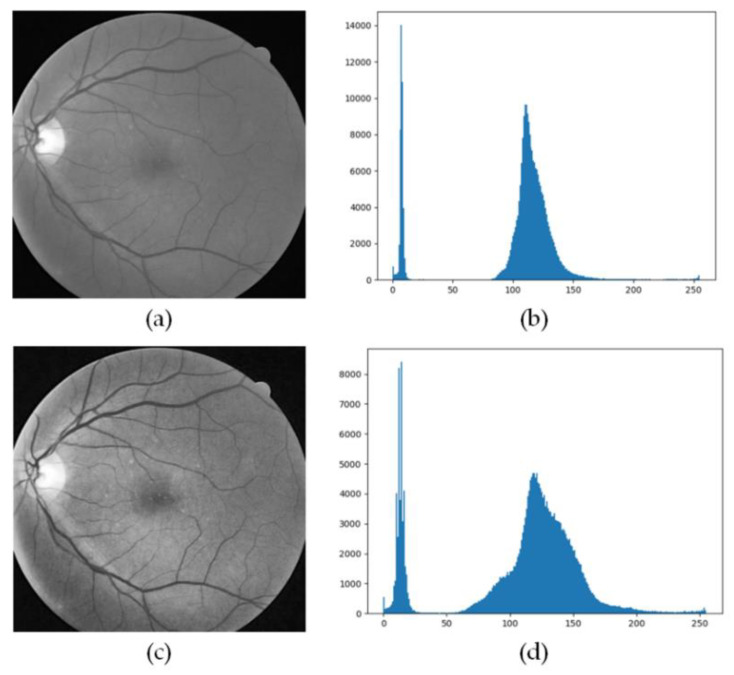
Comparison of results after CLAHE processing. (**a**) G channel image, (**b**) G channel histogram, (**c**) Image processed by CLAHE, (**d**) Histogram processed by CLAHE.

The image after processing the retinal blood vessel image using the CLAHE method is as seen in Figure 11. 

### 2.7. Experiment Setup

We used a PC equipped with an Intel Core i7-6700k, 4 GHz CPU (Intel, Penang, Malaysia) with 16 GB RAM (Samsung, Suzhou, China) and 6 GB of RTX2070S GPU (GIGABYTE, Taipei, Taiwan) for MSFFU-Net training. The operating system of the computer was 64-bit Win10. The structure of the network was implemented under the open source deep learning library TensorFlow with Pycharm implementation. In addition, Numpy (scientific computing library), some methods of image processing in OpenCV and some libraries in sklearn were used for image processing. 

In the process of model training, the stochastic gradient descent optimization algorithm was used to iteratively solve the parameters of the fundus retinal vascular image segmentation network. The initial learning rate was set to 0.001, which was changed to 0.1 times the current value every 20 epochs. The batch size was set to 20, and total training epochs to 100 [38]. It took about 60 h to train the network on this platform. 

### 2.8. Evaluation Metrics

In order to quantitatively evaluate the performance of the segmentation results of the proposed algorithm, four evaluation indicators were used in this paper named: Sensitivity (Sen), Specificity (Spe), Accuracy (Acc) and Area Under Curve (AUC) to evaluate the experimental results. Sensitivity is the ratio of the number of correctly detected retinal blood vessel pixels to the total number of blood vessel pixels. Specificity is the ratio of the number of correctly detected non-vessel pixels to the total number of non-vessel pixels. Accuracy is the ratio of the number of correctly detected blood vessels and background pixels to the total number of image pixels. The expressions of Se, Sp and Acc are defined as follows:

(10) $$Sensitivity=\frac{TP}{TP+FN}$$

(11) $$Specificity=\frac{TN}{FP+TN}$$

(12) $$Accuracy=\frac{TP+TN}{TP+FN+TN+FP}$$

Where TP, TN, FP and FN denote true positive, true negative, false positive and false negative, respectively. In this model, positive refers to blood vessels and negative refers to background. Therefore, they are the four kinds of retinal vessel segmentation results based on the fact that each pixel can be segmented correctly or incorrectly. TP represents vascular pixels are correctly detected as blood vessels; TN represents background pixels are correctly detected as background; FP represents background pixels are incorrectly detected as blood vessels; FN represents vascular pixels are incorrectly detected as background.

The ROC curve is an important method for measuring the comprehensive performance of Image semantic segmentation results. For the ROC curve, the horizontal axis is the FPR, representing the percentage of detected blood vessel pixels in all the blood vessel pixels. And the vertical axis is the TPR, denoting the false detection of background pixels into the proportion of blood vessels in the real background pixels. They can be written as:

(13) $$TPR=\frac{TP}{TP+FN}$$

(14) $$FPR=\frac{FP}{TN+FP}$$

The AUC value represents the area under the ROC curve. Its value ranges from 0 to 1. Condition AUC = 1, a perfect classifier; 0.5 < AUC < 1, better than random classifiers; 0 < AUC < 0.5, worse than the random classifier.

## 3. Results and Discussion

### 3.1. Comparison with U-Net Model

To show the performance of proposed model, we compared the retinal vessel segmentation results on DRIVE test dataset and STARE test dataset with the U-Net model. We did the analysis from both qualitative and quantitative perspectives. Accordingly, in the contrast experiments, the same experimental configuration, parameters, and number of training sets are used in the training of U-Net model. 

#### 3.1.1. Qualitative Analysis

**Figure 12:**
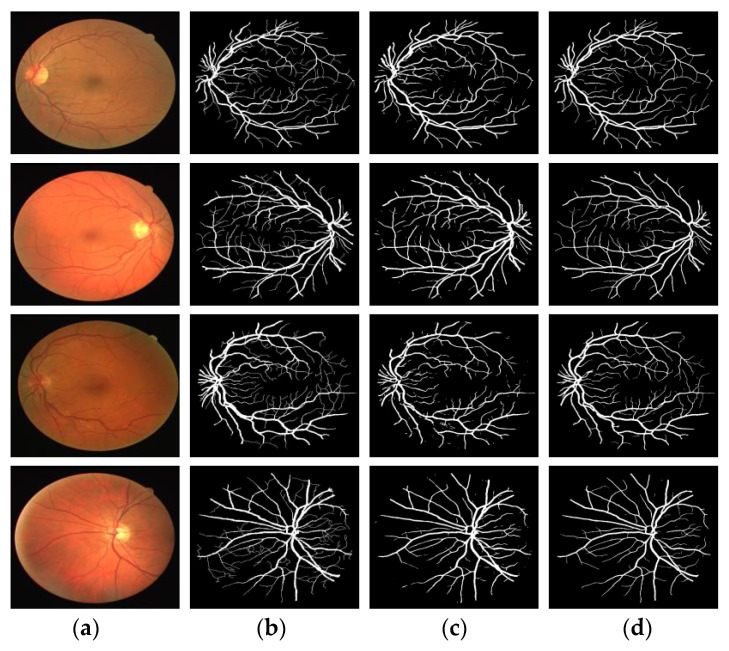
Comparison of segmentation result in DRIVE by proposed model and U-Net model. (**a**) Original image; (**b**) Ground truth; (**c**) Results by U-Net; (**d**) Results by MSFFU-Ne

**Figure 13:**
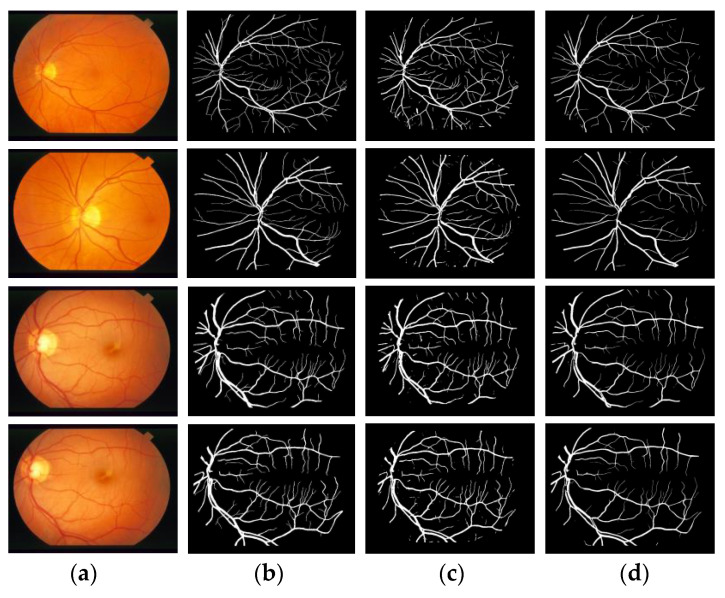
Comparison of segmentation result in STARE by proposed model and U-Net model. (**a**) Original image; (**b**) Ground truth; (**c**) Results by U-Net; (**d**) Results by MSFFU-Net.

Figure 12 and Figure 13 compare the segmentation results of two retinal blood vessel images from the DRIVE and STARE test datasets, respectively. In the two figures, column (a) shows the original retinal image; column (b) shows the ground truth; column (c) shows the segmentation results by U-Net model and column (d) shows the segmentation results by proposed model. The effectiveness of the proposed model was demonstrated, and the segmentation results are superior to the U-Net model.

**Figure 14:**
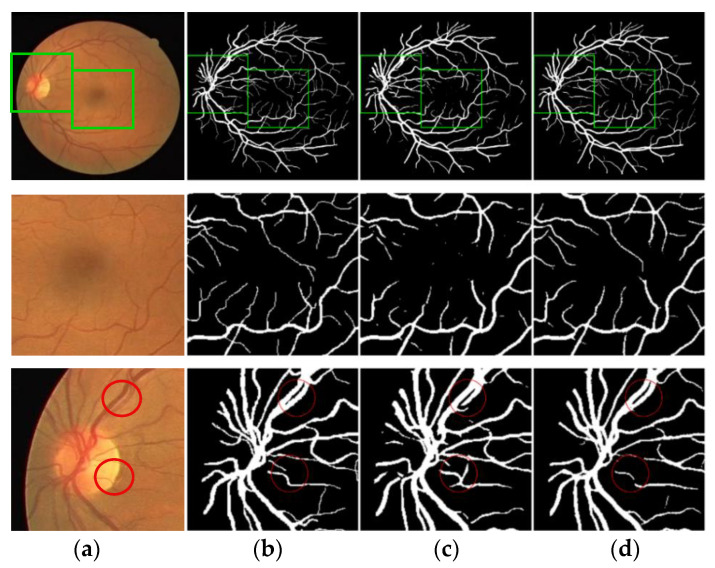
Comparison of the local segmentation results from DRIVE. (**a**) Original image; (**b**) Ground truth; (**c**) Results by U-Net; (**d**) Results by MSFFU-Net. The first row are the full retinal blood vessel images; the second row represents the tiny retinal vessels; the third row displays the densely packed blood vessels.

**Figure 15:**
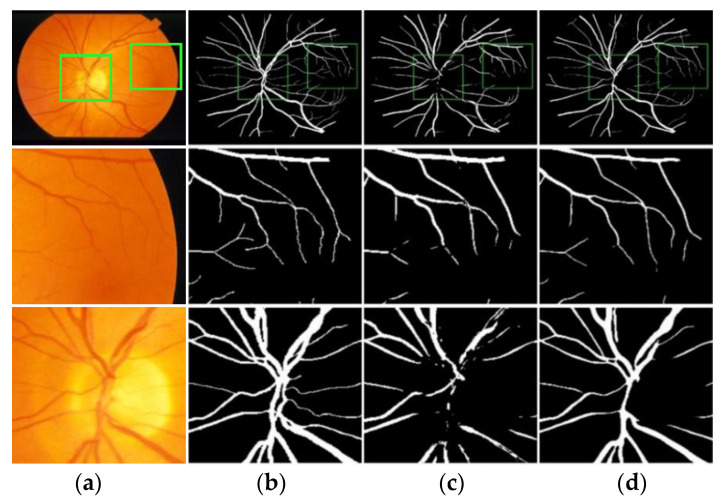
Comparison of the local segmentation results from STARE. (**a**) Original image; (**b**) Ground truth; (**c**) Results by U-Net; (**d**) Results by MSFFU-Net. The first row are the full retinal blood vessel images; the second row represents the tiny retinal vessels; the third row displays the densely packed blood vessels.

A set of segmentation results comparisons were selected from DRIVE and STARE, respectively, for local magnification analysis. Figure 14 and Figure 15 show the local segmentation results of the U-Net model and the MSFFU-Net model in the two datasets.

The first row of images represents the retinal original image, the manual segmentation result and the result images obtained by the two modes; the second row of images represents the tiny retinal vessel; the third row of images represents the densely packed blood vessels. The images of the second row and the third row are local details of the first row. Obviously, although U-Net model can detect most of the retinal blood vessels, the detection effectiveness of microvessels and dense vessels is poor. It is observable that the segmentation results by U-Net appears multiple incoherent vessels and mis-classification of background and vessel pixels. However, the MSFFU-Net model proposed in this paper has better classification performance and the ability to detect more tiny vessels. It proves that the feature fusion decoder structure applied max-pooling indices can recorde more accurately the retinal vascular edge and location information, and the multi-scale feature extraction encoder based on Inception module can make the thin tiny retinal blood vessel features more discriminative, which can present excellent segmentation performance [35]. Therefore, it also demonstrates that the proposed model has better performance on retinal blood vessels segmentation than the U-Net model. 

#### 3.1.2. Quantitative Analysis

The comparison of U-Net and proposed model based on evaluation metrics from DRIVE and STARE is shown in Table 1.

For both models, the values of Sen, Spe, Acc, and AUC using the proposed model in DRIVE dataset are higher than those when using the U-Net model. Only the Spe in STARE dataset is lower when using the proposed model compared to using the U-Net model. It demonstrates that the performance of the proposed model is superior to the U-Net model. 

### 3.2. Comparison with the State-of-the-Art Methods

**Figure 16:**
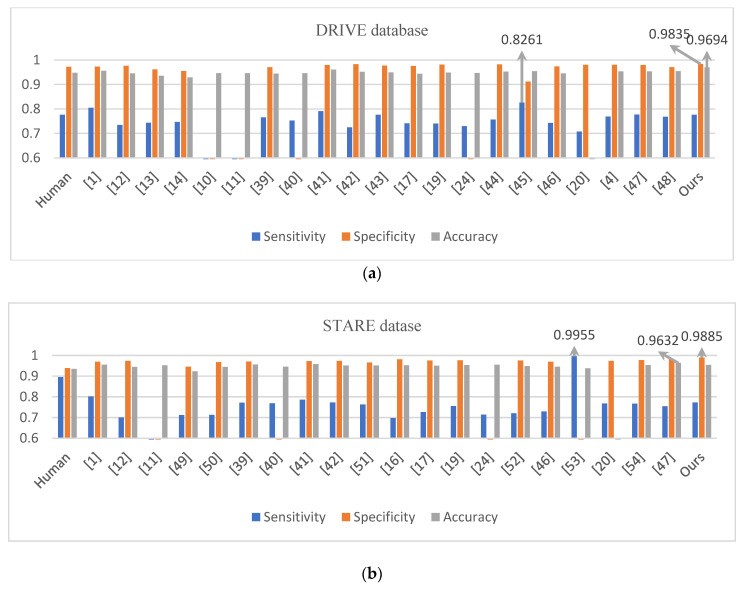
Comparison of segmentation results. (**a**) DRIVE (**b**) STARE.

To further demonstrate the performance for retinal vessel segmentation, we compared the proposed method with several other state-of-the-art methods using the DRIVE and STARE datasets, as shown in Table 2 and Table 3. Meanwhile, in order to show the advantages of the proposed method more intuitively, the comparison result is shown in Figure 16. In the DRIVE dataset, proposed method achieved a better result than human observer on all evaluation metrics. The sensitivity was 0.7762; the specificity was 0.9835; and the accuracy was 0.9694. Comparing evaluation indicators, most unsupervised methods are inferior to the method in this paper. However, the sensitivity value of the reference [1] and reference [39] is 0.0281 and 0.0145 higher than proposed method respectively. Khawaja et al. presented a directional multi-scale line detector technology in [1] for retinal blood vessel segmentation, mainly focusing on the tiny vessels that are most difficult to separate out. Khawaja et al. have devised a new strategy in [39] by introducing a denoiser that precedes the vessel segmentation step, which boosted the efficiency of Frangi filter detection tiny vessel. Compared with the other two evaluation parameters, the methods proposed in this paper are superior to [1] and [39]. Moreover, the proposed method ranks first among supervised methods in terms of specificity and accuracy, which proved that it could better classify background pixels and blood vessel pixels. In terms of sensitivity, the value of the method in this paper is 0.0499, 0.0001 and 0.001 lower than Maninis, Liskowsk and Hu, respectively. The resulting image segmented with Maninis et al.’s method contains a lot of noise, and the segmented vessels are thicker than real. 

Some background pixels are also detected as vascular pixels, so that the sensitivity is high and the specificity is low. However, the MSFFU-Net model uses the multi-scale feature extraction encoder module to more fully capture deep features at different scales, and the receiving field of feature maps of is larger, which can segment the pathological regions well. We used the skip connection and max-pooling index to alleviate the difficulty of decoding to recover microvessels, so that the segmented retinal vessel image contains less pathology and the segmentation results are more accurate. Image preprocessing and cost-sensitive loss functions also contributed significantly to this result. As can be seen from Table 3 and Figure 16b, the specificity of the method for the STARE dataset are highest in those listed methods, and the sensitivity value of the proposed method in this paper is 0.029, 0.0139 and 0.2234 lower than that in Ref. [1], Ref. [39] and Xie et al.’s method respectively. The HED algorithm in the Xie et al.’s method can detect edge information with a small receptive field. Therefore, due to the influence of the network model they proposed, the algorithm segmented a larger blood vessel size, sometimes resulting in a blurred blood vessel segmentation image. As for the accuracy, the value of the proposed method is 0.0008, 0.0026, 0.0046, 0.0008 and 0.0095 lower than that of [1], Azzopardi, [39], Fu and Hu, respectively. However, the sensitivity or specificity of the method in this paper are higher. Therefore, it can be seen from the evaluation metrics in Table 2 and Table 3 and Figure 16 that the performance of the proposed method in this paper is superior.

## 4. Conclusions

In this study, we propose a multi-scale feature fusion retinal vessel segmentation method based on U-Net, which integrates data augmentation, data preprocessing, and a MSFFU-Net model. The model introduced the inception structure into the multi-scale feature extraction encoder part of the improved network, and we optimized the basic inception structure, reducing the training parameters of the model. The feature maps obtained by the inception module of the multi-scale convolution kernel designed in this paper have different sizes of receptive fields, which can have a better detection effect on the vascular structures of different sizes in the retinal blood vessel image and reduce the inaccurate segmentation of tiny vessels. The max-pooling index was applied during the upsampling process in the feature fusion decoder part, precisely retaining the location information of the object features, and obtaining a clearer contour of retinal blood vessels in the segmentation results. The multiscale skip connections architecture in the model transferred the detailed features from the encoder to the decoder. In addition, we designed a cost-sensitive loss function based on the Dice coefficient and cross entropy, which alleviated the problem of imbalance between the number of blood vessel pixels and the background pixels in the fundus retina image. Four transformations were used as data augmentation strategies to improve the generalization ability of the proposed method. Then, the images were preprocessed using the CLAHE algorithm. The stochastic gradient descent (SGD) optimization algorithm was used to iteratively solve the network parameters in the process of model training. We adopted the DRIVE dataset and STARE datasets for training and testing of the proposed framework, and Sen, Spe, Acc and AUC were used as evaluation metrics. The experimental results demonstrate the superiority of the presented method to other supervised and unsupervised learning methods, therefore, the method proposed in this paper showed its competitive results.

Although the inception blocks, storage of pooling indices, multiscale skip connections and the combination of dice and loss function are not the ideas we proposed, we are the first to integrate them into a system retinal vessel segmentation and add our own improvements. As mentioned earlier, these modules play a very important role in the multi-scale feature fusion method based on U-Net for retinal vessel segmentation proposed in this paper. In the future, we plan to improve the image preprocessing algorithm to achieve better segmentation performance on the STARE dataset. Furthermore, the attention mechanism will be added so that the extracted feature maps have different weights, which improves the utilization of feature information and more accurately locates the contour area of the fundus.

## Figures and Tables

**Table 1:** Comparison of U-Net model and MSFFU-Net model based on evaluation metrics.

Model	DRIVE				STARE			
	Sen	Spe	Acc	AUC	Sen	Spe	Acc	AUC
U-Net	0.7758	0.9755	0.9500	0.9742	0.7838	0.9780	0.9535	0.9673
Proposed model	0.7762	0.9835	0.9694	0.9790	0.7721	0.9885	0.9537	0.9680

**Table 2:** Comparison of proposed method with other methods in DRIVE database.

Type	Method	Sensitivity	Specificity	Accuracy
	Human observer	0.7760	0.9724	0.9472
Unsupervised methods	Khawaja [1]	0.8043	0.9730	0.9553
	Mendonca [12]	0.7344	0.9764	0.9452
	Espona [13]	0.7436	0.9615	0.9352
	Vlachos [14]	0.7468	0.9551	0.9285
	Miri [10]	–	–	0.9458
	Wang [11]	–	–	0.9461
	Azzopardi [40]	0.7655	0.9704	0.9442
	Wang [41]	0.7527	–	0.9457
	Khawaja [39]	0.7907	0.9790	0.9603
	Roychowdhury [42]	0.7250	0.9830	0.9520
Supervised Methods	Liskowsk [43]	0.7763	0.9768	0.9495
	You [17]	0.7410	0.9751	0.9434
	Fraz [19]	0.7406	0.9807	0.9480
	Fu [24]	0.7294	–	0.9470
	Li [44]	0.7569	0.9816	0.9527
	Maninis [45]	0.8261	0.9115	0.9541
	Chen [46]	0.7426	0.9735	0.9453
	Orlando [20]	0.7079	0.9802	–
	Dasgupta [4]	0.7691	0.9801	0.9533
	Hu [47]	0.7772	0.9793	0.9533
	Na [48]	0.7680	0.9700	0.9540
	Proposed method	0.7762	0.9835	0.9694

**Table 3:** Comparison of proposed method with other methods in STARE database.

Type	Method	Sensitivity	Specificity	Accuracy
	Human observer	0.8952	0.9384	0.9349
Unsupervised methods	Khawaja [1]	0.8011	0.9694	0.9545
	Mendonca [12]	0.6996	0.9730	0.9440
	Wang [11]	–	–	0.9521
	Aguiree [49]	0.7116	0.9454	0.9231
	Soomro [50]	0.7130	0.9680	0.9440
	Azzopardi [40]	0.7716	0.9701	0.9563
	Wang [41]	0.7686	–	0.9451
	Khawaja [39]	0.7860	0.9725	0.9583
	Roychowdhury [42]	0.7720	0.9730	0.9510
	Mapayi [51]	0.7626	0.9657	0.9510
Supervised methods	Staal [16]	0.6970	0.9810	0.9520
	You [17]	0.7260	0.9756	0.9497
	Fraz [19]	0.7548	0.9763	0.9534
	Fu [24]	0.7140	–	0.9545
	Soares [52]	0.7200	0.9750	0.9480
	Chen [46]	0.7295	0.9696	0.9449
	Xie [53]	0.9955	0.5555	0.9378
	Orlando [20]	0.7680	0.9738	–
	Xia [54]	0.7670	0.9770	0.9530
	Hu [47]	0.7543	0.9814	0.9632
	Proposed method	0.7721	0.9885	0.9537
